# Focus on prevention, diagnosis and treatment of hypertension in children and adolescents

**DOI:** 10.1186/1824-7288-39-20

**Published:** 2013-03-19

**Authors:** Amedeo Spagnolo, Marco Giussani, Amalia Maria Ambruzzi, Mario Bianchetti, Silvio Maringhini, Maria Chiara Matteucci, Ettore Menghetti, Patrizia Salice, Loredana Simionato, Mirella Strambi, Raffaele Virdis, Simonetta Genovesi

**Affiliations:** 1 ISFOL, Istituto per gli Affari Sociali, Rome, Italy; 2 Pediatra di Famiglia, Progetto PAB (Pressione Arteriosa Bambino), Milan, Italy; 3 Pediatra-nutrizionista, Ospedale Pediatrico Bambino Gesù, Rome, Italy; 4 Department of Pediatrics, Mendrisio and Bellinzona Hospitals, Switzerland, and University of Bern, Switzerland, Bern, Switzerland; 5 Unità Operativa Complessa Nefrologia Pediatrica, Ospedale dei Bambini “G. Di Cristina” A.R.N.A. S. “Civico, Di Cristina e Benefratelli”, Palermo, Italy; 6 Divisione di Nefrologia Pediatrica Ospedale Pediatrico Bambino Gesù, IRCCS, Società Italiana di Nefrologia Pediatrica, Rome, Italy; 7 Pediatra-nutrizionista, Università”La Sapienza”, Rome, Italy; 8 Cardiologia Pediatrica UO Cardiologia Fondazione Policlinico Ca Granda IRCCS, Progetto CHild, Milan, Italy; 9 Pediatra di Famiglia, Progetto CHild, Milan, Italy; 10 Dipartimento di Pediatria, Ostetricia e Medicina della Riproduzione, Università di Siena, Siena, Italy; 11 Dipartimento Età Evolutiva, Clinica Pediatrica, Università di Parma, Parma, Italy; 12 Clinica Nefrologica e Dipartimento di Medicina Clinica e Prevenzione Università di Milano Bicocca. Progetto PAB (Pressione Arteriosa Bambino), Società Italiana Ipertensione Arteriosa, Milan, Italy

**Keywords:** Blood pressure, Children, Hypertension, Obesity, Overweight, Prevention, Physical activity, Salt intake

## Abstract

The European Society of Hypertension has recently published its recommendations on prevention, diagnosis and treatment of high blood pressure in children and adolescents. Taking this contribution as a starting point the Study Group of Hypertension of the Italian Society of Pediatrics together with the Italian Society of Hypertension has conducted a reappraisal of the most recent literature on this subject. The present review does not claim to be an exhaustive description of hypertension in the pediatric population but intends to provide Pediatricians with practical and updated indications in order to guide them in this often unappreciated problem.

This document pays particular attention to the primary hypertension which represents a growing problem in children and adolescents. Subjects at elevated risk of hypertension are those overweight, with low birth weight and presenting a family history of hypertension. However, also children who do not present these risk factors may have elevated blood pressure levels. In pediatric age diagnosis of hypertension or high normal blood pressure is made with repeated office blood pressure measurements that show values exceeding the reference values. Blood pressure should be monitored at least once a year with adequate methods and instrumentation and the observed values have to be interpreted according to the most updated nomograms that are adjusted for children’s gender, age and height. Currently other available methods such as ambulatory blood pressure monitoring and home blood pressure measurement are not yet adequately validated for use as diagnostic instruments. To diagnose primary hypertension it is necessary to exclude secondary forms. The probability of facing a secondary form of hypertension is inversely proportional to the child’s age and directly proportional to blood pressure levels. Medical history, clinical data and blood tests may guide the differential diagnosis of primary versus secondary forms. The prevention of high blood pressure is based on correct lifestyle and nutrition, starting from childhood age. The treatment of primary hypertension in children is almost exclusively dietary/behavioral and includes: a) reduction of overweight whenever present b) reduction of dietary sodium intake c) increase in physical activity. Pharmacological therapy will be needed rarely and only in specific cases.

## Introduction

Before reference nomograms for blood pressure in childhood were available, the diagnosis of hypertension was made only in the presence of highly elevated blood pressure levels. Practically only the most severe secondary forms were diagnosed. Through the publication of the first reference values [1] is has been possible to reveal that there is a large number of children with blood pressure levels above the normal range and that this condition can be almost completely ascribed to primary hypertension. The rise in the prevalence of overweight children and the increased survival rate of subjects with a very low birth weight may predict that the progression of hypertension prevalence in pediatric subjects will continue to aggravate. In 2009 the European Society of Hypertension published recommendations for the management of hypertension in children and adolescents [2]. Longitudinal studies have shown that quite frequently children with elevated blood pressure levels are destined to become hypertensive adults [3]. Better diagnostic techniques for detecting subclinical organ damage have allowed us to become aware that even in childhood high blood pressure may be accompanied by structural and functional changes in some organs. The Italian Society of Pediatrics and the Italian Society of Hypertension aim at providing the recommendations that are suitable for the Italian health care situation. These indications do not claim to be an exhaustive description of the problems of hypertension in the years of growth, but they intend to provide Pediatricians and Family Doctors with updated recommendations on prevention, diagnosis and treatment in order to prevent organ damage that might emerge if hypertension is not properly treated. This task however is not easy as there are no observational studies in children on the relationship between blood pressure values and cardiovascular events that may arise many years later. Besides, large intervention trials in children are lacking at the moment.

### Definition

Hypertension in children is defined using a statistical criterion, the limit being the 95^th^ percentile of the distribution of the systo-diastolic blood pressure values, according to gender, age and height. In order to conform to the adult terminology pre-hypertension or high-normal pressure hypertension is defined as blood pressure values consistently above or equal to the 90^th^ percentile, but lower than the 95^th^[4] (Table 1).

**Table 1:** Definition and classification of hypertension in children and adolescents

**Category**	**Systolic or diastolic blood pressure percentile**
Normal	< 90^th^
Pre-hypertension	≥ 90^th^ and < 95^th^
	≥ 120/80 mmHg independently of the 90^th^ percentile value in adolescents
Stage 1 hypertension	≥ 95^th^ and < 99^th^ + 5 mmHg
Stage 2 hypertension	≥ 99^th^ + 5 mmHg

4° Report on the Diagnosis, Evaluation and Treatment of High Blood Pressure in Children and Adolescents. *Pediatrics* 2004 [4].

**Figure 1:**
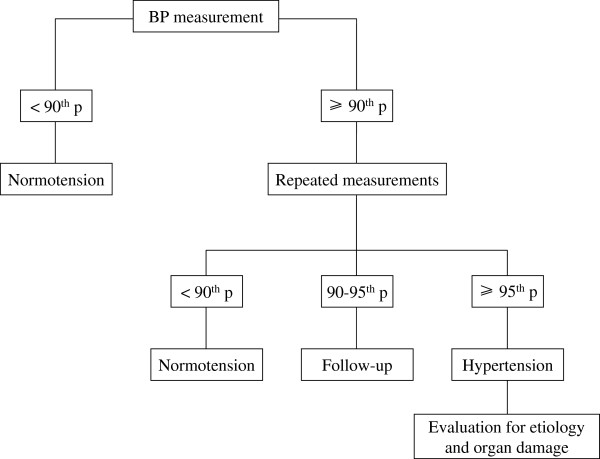
Diagnostic algorithm of hypertension in children and adolescents.

Figure 1 shows the algorithm to be used for a correct diagnosis of hypertension.

### Epidemiology

Prevalence and new diagnoses of hypertension in children and adolescents are increasing [5]. Numerous population studies indicate that a hypertensive condition in childhood raises the probability of being hypertensive in adulthood [3]. In the first years of childhood secondary forms prevail whereas with increasing age primary forms of hypertension become most frequent. Blood pressure values increase progressively until the age of 17–18 years when adult values are reached. This increase is most rapid during the first weeks of life and during puberty. Blood pressure values are correlated with gender, height and body mass. Obesity represents a strong risk factor for the development of child hypertension. There are no sufficient data on the role of ethnicity, but many studies demonstrate that Afro-American children have higher blood pressure values than Caucasian children [5,6]. The heritability of childhood hypertension is estimated to be about 50% [7,8]. Eighty-six percent of adolescents with primary hypertension have a positive family history for hypertension [9]. Breastfeeding is associated with lower blood pressure levels in childhood [10-12].

### Methodology for blood pressure recording in children and reference values

For correct blood pressure recording it is necessary to conform the measuring procedure to the method used for the construction of the reference tables. The children should be calm and relaxed, seated with their back supported and their right arm resting at heart level. The cuff should be of the appropriate size for the children’s upper arm. Small cuffs tend to overestimate while large cuffs underestimate. The width of the inflatable cuff should be 40% of the arm circumference at a point midway between the olecranon and the acromion. A practical way for estimating the appropriateness of the size is to place the short segment of the cuff on the central part of the child’s arm and assure that the arm is encircled by little less than 50%. In case of doubt it is advised to use the larger cuff. The cuff bladder length should cover 80% to 100% of the circumference of the arm. The stethoscope should be placed over the brachial artery pulse, proximal and medial to the cubital fossa, and below the bottom edge of the cuff. The bladder should be inflated up to 20 mmHg beyond the disappearance of the radial pulse and then deflated at a rate of 2–3 mmHg per second. Systolic blood pressure is defined by the first Korotkoff sound (K1; appearance of the pulse), whereas diastolic blood pressure coincides with the disappearance of the pulse (K5). If Korotkoff sounds do not disappear the muffling of the sounds (K4) should be considered for diastolic blood pressure. At least three measurements performed on different occasions are necessary for the diagnosis of hypertension. Currently the mercury sphygmomanometers have been proscribed due to their toxicity. The use of oscillometric devices in children may potentially be a source of errors. The Internet site http://www.dableducational.org reports the oscillometric devices that have been validated by the scientific societies. A diagnosis of hypertension based on an oscillometric measurement should be confirmed by an auscultatory method, using a non-mercury manometer (aneroid). The aneroid devices need to be calibrated every six months. Children above 3 years of age should have their blood pressure measured every year on the occasion of the periodic visits. In all children including the youngers ones blood pressure should be measured under special circumstances that increase the risk for hypertension: intensive neonatal care, renal disease, treatment with drugs known to increase blood pressure, evidence of elevated intracranial pressure.

Almost all the studies on pediatric populations have used US nomograms for reference. The Recommendations of the European Society of Hypertension refer to these tables as well (Tables 2 and 3) [4]. It would be advisable to consult these nomograms using the North-American reference values of height percentile. (http://www.cdc.gov/growthcharts/clinical_charts). It has to be underlined that the American nomograms have been obtained by the auscultatory method.

**Table 2:** Blood pressure percentiles for boys by age and height

		**Systolic (mmHg) percentile of height**							**Diastolic (mmHg) percentile of height**						
**Age (years)**	**BP percentile**	**5th**	**10th**	**25th**	**50th**	**75th**	**90th**	**95th**	**5th**	**10th**	**25th**	**50th**	**75th**	**90th**	**95th**
1	90th	94	95	97	99	100	102	103	49	50	51	52	53	53	54
	95th	98	99	101	103	104	106	106	54	54	55	56	57	58	58
	99th	105	106	108	110	112	113	114	61	62	63	64	65	66	66
2	90th	97	99	100	102	104	105	106	54	55	56	57	58	58	59
	95th	101	102	104	106	108	109	110	59	59	60	61	62	63	63
	99th	109	110	111	113	115	117	117	66	67	68	69	70	71	71
3	90th	100	101	103	105	107	108	109	59	59	60	61	62	63	63
	95th	104	105	107	109	110	112	113	63	63	64	65	66	67	67
	99th	111	112	114	116	118	119	120	71	71	72	73	74	75	75
4	90th	102	103	105	107	109	110	111	62	63	64	65	66	66	67
	95th	106	107	109	111	112	114	115	66	67	68	69	70	71	71
	99th	113	114	116	118	120	121	122	74	75	76	77	78	78	79
5	90th	104	105	106	108	110	111	112	65	66	67	68	69	69	70
	95th	108	109	110	112	114	115	116	69	70	71	72	73	74	74
	99th	115	116	118	120	121	123	123	77	78	79	80	81	81	82
6	90th	105	106	108	110	111	113	113	68	68	69	70	71	72	72
	95th	109	110	112	114	115	117	117	72	72	73	74	75	76	76
	99th	116	117	119	121	123	124	125	80	80	81	82	83	84	84
7	90th	106	107	109	111	113	114	115	70	70	71	72	73	74	74
	95th	110	111	113	115	117	118	119	74	74	75	76	77	78	78
	99th	117	118	120	122	124	125	126	82	82	83	84	85	86	86
8	90th	107	109	110	112	114	115	116	71	72	72	73	74	75	76
	95th	111	112	114	116	118	119	120	75	76	77	78	79	79	80
	99th	119	120	122	123	125	127	127	83	84	85	86	87	87	88
9	90th	109	110	112	114	115	117	118	72	73	74	75	76	76	77
	95th	113	114	116	118	119	121	121	76	77	78	79	80	81	81
	99th	120	121	123	125	127	128	129	84	85	86	87	88	88	89
10	90th	111	112	114	115	117	119	119	73	73	74	75	76	77	78
	95th	115	116	117	119	121	122	123	77	78	79	80	81	81	82
	99th	122	123	125	127	128	130	130	85	86	86	88	88	89	90
11	90th	113	114	115	117	119	120	121	74	74	75	76	77	78	78
	95th	117	118	119	121	123	124	125	78	78	79	80	81	82	82
	99th	124	125	127	129	130	132	132	86	86	87	88	89	90	90
12	90th	115	116	118	120	121	123	123	74	75	75	76	77	78	79
	95th	119	120	122	123	125	127	127	78	79	80	81	82	82	83
	99th	126	127	129	131	133	134	135	86	87	88	89	90	90	91
13	90th	117	118	120	122	124	125	126	75	75	76	77	78	79	79
	95th	121	122	124	126	128	129	130	79	79	80	81	82	83	83
	99th	128	130	131	133	135	136	137	87	87	88	89	90	91	91
14	90th	120	121	123	125	126	128	128	75	76	77	78	79	79	80
	95th	124	125	127	128	130	132	132	80	80	81	82	83	84	84
	99th	131	132	134	136	138	139	140	87	88	89	90	91	92	92
15	90th	122	124	125	127	129	130	131	76	77	78	79	80	80	81
	95th	126	127	129	131	133	134	135	81	81	82	83	84	85	85
	99th	134	135	136	138	140	142	142	88	89	90	91	92	93	93
16	90th	125	126	128	130	131	133	134	78	78	79	80	81	82	82
	95th	129	130	132	134	135	137	137	82	83	83	84	85	86	87
	99th	136	137	139	141	143	144	145	90	90	91	92	93	94	94
17	90th	127	128	130	132	134	135	136	80	80	81	82	83	84	84
	95th	12	132	134	136	138	139	140	84	85	86	87	87	88	89
	99th	139	140	141	143	145	146	147	92	93	93	94	95	96	97

4° Report on the Diagnosis, Evaluation and Treatment of High Blood Pressure in Children and Adolescents. *Pediatrics* 2004 [4].

**Table 3:** Blood pressure percentiles for girls by age and height

		**Systolic (mmHg0 percentile of height**							**Diastolic (mmHg) percentile of height**						
**Age (years)**	**BP percentile**	**5th**	**10th**	**25th**	**50th**	**75th**	**90th**	**95th**	**5th**	**10th**	**25th**	**50th**	**75th**	**90th**	**95th**
1	90th	97	97	98	100	101	102	103	52	53	53	54	55	55	56
	95th	100	101	102	104	105	106	107	56	57	57	58	59	59	60
	99th	108	108	109	111	112	113	114	64	64	65	65	66	67	67
2	90th	98	99	100	101	103	104	105	57	58	58	59	60	61	61
	95th	102	103	104	105	107	108	109	64	62	62	63	64	65	65
	99th	109	110	111	112	114	115	116	69	69	70	70	71	72	72
3	90th	100	100	102	103	104	106	106	61	62	62	63	64	64	65
	95th	104	104	105	107	108	109	110	65	66	66	67	68	68	69
	99th	111	111	113	114	115	116	117	73	73	74	74	75	76	76
4	90th	101	102	103	104	106	107	108	64	64	65	66	67	67	68
	95th	105	106	107	108	110	111	112	68	68	69	70	71	71	72
	99th	112	113	114	115	117	118	119	76	76	76	77	78	79	79
5	90th	103	103	105	106	107	109	109	66	67	67	68	69	69	70
	95th	107	107	108	110	111	112	113	70	71	71	72	73	73	74
	99th	114	114	116	117	118	120	120	78	78	79	79	80	81	81
6	90th	104	105	106	108	109	110	111	68	68	69	70	70	71	72
	95th	108	109	110	111	113	114	115	72	72	73	74	74	75	76
	99th	115	116	117	119	120	121	122	80	80	80	81	82	83	83
7	90th	106	107	108	109	111	112	113	69	70	70	71	72	72	73
	95th	110	111	112	113	115	116	116	73	74	74	75	76	76	77
	99th	117	118	119	120	122	123	124	81	81	82	82	83	84	84
8	90th	108	109	110	111	113	114	114	71	71	71	72	73	74	74
	95th	112	112	114	115	116	118	118	75	75	75	76	77	78	78
	99th	119	120	121	122	123	125	125	82	82	83	83	84	85	86
9	90th	110	110	112	113	114	116	116	72	72	72	73	74	75	75
	95th	114	114	115	117	118	119	120	76	76	76	77	78	79	79
	99th	121	121	123	124	125	127	127	83	83	84	84	85	86	87
10	90th	112	112	114	115	116	118	118	73	73	73	74	75	76	76
	95th	116	116	117	119	120	121	122	77	77	77	78	79	80	80
	99th	123	123	125	126	127	129	129	84	84	85	86	86	87	88
11	90th	114	114	116	117	118	119	120	74	74	74	75	76	77	77
	95th	118	118	119	121	122	123	124	78	78	78	79	80	81	81
	99th	125	125	126	128	129	130	131	85	85	86	87	87	88	89
12	90th	116	116	117	119	120	121	122	75	758	75	76	77	78	78
	95th	119	120	121	123	124	125	126	79	79	79	80	81	82	82
	99th	127	127	128	130	131	132	133	86	86	87	88	88	89	90
13	90th	117	118	119	121	122	123	124	76	76	76	77	78	79	79
	95th	121	122	123	124	126	127	128	80	80	80	81	82	83	83
	99th	128	129	130	132	133	134	135	87	87	88	89	89	90	91
14	90th	119	120	121	122	124	125	125	77	77	77	78	79	80	80
	95th	123	123	125	126	127	129	129	81	81	81	82	83	84	84
	99th	130	131	132	133	135	136	136	88	88	89	90	90	91	92
15	90th	120	121	122	123	125	126	127	78	78	78	79	80	81	81
	95th	124	125	126	127	129	130	131	82	82	82	83	84	85	85
	99th	131	132	133	134	136	137	138	89	89	90	91	91	92	93
16	90th	121	122	123	124	126	127	128	78	78	79	80	81	81	82
	95th	125	126	127	128	130	131	132	82	82	83	84	85	85	86
	99th	132	133	134	135	137	138	139	90	90	90	91	92	93	93
17	90th	122	122	123	125	126	127	128	78	79	79	80	81	81	82
	95th	125	126	127	129	130	131	132	82	83	83	84	85	85	86
	99th	133	133	134	136	137	138	139	90	90	91	91	92	93	93

4° Report on the Diagnosis, Evaluation and Treatment of High Blood Pressure in Children and Adolescents. *Pediatrics* 2004 [4].

Twenty-hour hour blood pressure monitoring (Ambulatory Blood Pressure Monitoring) is validated and used in adults for the diagnosis of hypertension. It allows to identify “white coat hypertension” (elevated office blood pressure values and normal Ambulatory Blood Pressure Monitoring values) and “masked hypertension” (normal office blood pressure values and elevated Ambulatory Blood Pressure Monitoring values). This technique can also identify subjects with or without reduced physiological day-night blood pressure variations. In children the use of Ambulatory Blood Pressure Monitoring has significant limitations due to the lack of reference values that have been validated in sufficiently large populations. The only existing nomograms (recommended both by the American Heart Association and the European Society of Hypertension) refer to a study that is based on a relatively small number of subjects. The study provides the values corresponding to the 75^th^, 90^th^ e 95^th^ percentile of the mean daytime and nighttime blood pressure by gender and in accordance with age and height, displayed separately however (Tables 4 and 5) [13].

**Table 4:** Reference values of 24-h ambulatory blood pressure by age and gender

**Age (years)**	**Boys**						**Girls**					
	**Day**			**Night**			**Day**			**Night**		
	**75th**	**90th**	**95th**	**75th**	**90th**	**95th**	**75th**	**90th**	**95th**	**75th**	**90th**	**95th**
5	116/76	120/79	123/81	99/59	103/62	106/65	114/77	118/80	121/82	100/81	105/66	108/69
6	116/76	121/79	124/81	100/59	105/63	108/66	155/77	120/80	122/82	101/61	106/65	110/68
7	117/76	122/80	125/82	101/60	106/64	110/67	116/77	121/80	123/82	102/60	107/65	111/67
8	117/76	122/80	125/82	102/60	108/64	111/67	117/76	122/80	124/82	103/60	108/64	112/67
9	118/76	123/80	126/82	103/60	109/64	112/67	118/76	122/80	125/82	103/59	109/64	112/6
10	119/76	124/80	127/82	104/60	110/64	113/67	119/76	123/79	126/81	104/59	110/64	113/67
11	121/76	126/80	129/82	105/60	111/64	115/67	120/76	120/79	127/81	105/59	110/63	114/66
12	123/76	128/80	132/82	107/60	113/64	116/67	121/76	125/80	128/82	105/59	110/63	114/66
13	126/76	131/80	1365/82	109/60	115/64	119/67	122/77	126/80	129/82	106/59	111/63	114/66
14	129/77	134/80	138/82	112/61	118/64	121/67	123/77	127/80	130/82	106/59	111/63	114/65
15	132/77	137/81	141/83	114/61	120/64	123/66	124/77	128/80	130/82	107/59	111/63	114/65
16	135/78	140/81	144/84	117/61	123/64	126/66	124/77	129/77	131/82	107/59	111/63	114/65

Wühl E et al., German Working Group on Pediatric Hypertension. “Distribution of 24-h ambulatory blood pressure in children: normalized reference values and role of body dimensions”. *J Hypertens* 2002; 20:1995–2007 [13].

**Table 5:** Reference values of 24-h ambulatory blood pressure by height and gender

**Height (cm)**	**Boys**						**Girls**					
	**Day**			**Night**			**Day**			**Night**		
	**75th**	**90th**	**95th**	**75th**	**90th**	**95th**	**75th**	**90th**	**95th**	**75th**	**90th**	**95th**
120	116/77	111/80	125/82	99/58	103/61	106/63	114/77	118/80	120/82	99/60	103/63	106/65
125	117/76	122/80	125/82	100/58	105/61	108/63	115/77	119/80	121/82	100/60	104/63	107/66
130	117/76	122/80	126/82	101/59	106/62	110/64	116/76	120/80	122/82	101/59	106/63	108/66
135	117/76	123/80	126/82	102/59	108/63	111/65	116/76	120/80	123/82	102/59	107/63	109/66
140	118/76	123/80	126/82	104/60	109/63	113/65	117/76	121/80	124/82	103/59	108/63	110/66
145	119/76	124/79	127/81	105/60	111/64	144/66	11876	123/80	125/82	103/59	109/63	112/66
150	120/76	125/79	128/81	106/60	112/64	116/66	119/76	124/80	127/82	104/59	110/63	113/66
155	122/76	127/79	130/81	107/60	113/64	117/66	121/76	125/80	128/82	106/59	111/63	114/66
160	124/76	129/79	133/81	108/60	144/64	188/66	122/76	126/80	129/82	106/59	111/63	114/66
165	126/76	132/80	135/82	110/60	116/64	119/66	123/77	127/80	130/82	107/59	112/63	114/66
170	128/77	134/80	138/82	112/61	117/64	121/66	124/77	128/80	131/82	108/61	112/67	115/71
175	130/77	136/81	140/83	113/61	119/64	112/66	125/78	129/81	131/82	109/59	113/63	115/66
180	132/77	138/81	142/83	115/61	120/64	124/66	N/A	N/A	N/A	N/A	N/A	N/A
185	134/78	140/81	144/84	116/61	122/64	125/66	N/A	N/A	N/A	N/A	N/A	N/A

Wühl E et al., German Working Group on Pediatric Hypertension. “Distribution of 24-h ambulatory blood pressure in children: normalized reference values and role of body dimensions”. *J Hypertens* 2002; 20:1995–2007 [3].

A new and important chapter in blood pressure monitoring is the self-measurement of blood pressure at home. Even in this case available data from children are scanty. In any way reference values have been suggested derived from a study on about 800 subjects. Correct self-measurement requires two measurements within a few minutes, performed in the morning and in the evening for 3 consecutive days (Table 6) [14].

**Table 6:** Reference values of home blood pressure by height and gender

**Height (cm)**	**Boys**			**Girls**		
	***N***	**50th**	**95th**	***N***	**50th**	**95th**
120-129	23	105/64	119/76	36	101/64	119/74
130-139	51	108/64	101/77	51	103/64	120/76
140-149	39	110/65	125/77	61	105/65	122/77
150-159	41	112/65	126/78	71	108/66	123/77
160-169	45	115/65	123/78	148	110/66	124/78
170-179	91	117/66	132/78	46	112/66	125/79
180-189	57	121/67	134/79	7	114/67	128/80

Stergiou GS et al. “Home blood pressure normalcy in children and adolescents: the Arsakeion School study”. *J Hypertens* 2007; 25:1375–1379 [14].

### Monitoring of organ damage

Hypertension is one of the main risk factors for the development of cardiac, cerebrovascular and renal diseases. It represents an important pathophysiological substrate for the development of atherosclerosis and consequent organ damage. Once hypertension has been diagnosed it is important to determine the presence of organ damage for the stratification of cardiovascular risk. Major attention should be paid to heart, arteries, kidney, nervous system and retina.

### Heart and vessels

Left ventricular hypertrophy which is associated with cardiac disease and mortality in adults is the organ damage that has been most documented in hypertensive children and adolescents. There are little data regarding the relationship between childhood hypertension and adult cardiovascular risk [15,16]. It has however been demonstrated that in children both physiological and pathological increases in blood pressure progressively modify the geometry of the left ventricle causing a significant increase in its wall thicknesses [17,18] and that cardiac mass is already subject to change during the early hypertensive stages [19] and associated with 24h systolic blood pressure [20]. Both the rate of development and the rate of regression of left ventricular hypertrophy are inversely correlated with age [21]. In hypertensive subjects with left ventricular hypertrophy normalization of blood pressure determines a echocardiographically detectable normalization of ventricle mass and functional parameters, but cardiovascular risk still remains elevated compared to normotensive subjects [22]. Therefore it is important to identify subjects with hypertension before left ventricular hypertrophy develops. In children the complex relationship between growth of the heart and of the body as a whole complicates the indexation of cardiac mass, especially in the lowest age ranges. Various methods for indexation have been proposed for pediatric patients. Ventricular mass can be calculated echocardiographically using the Devereux equation [23] indexed for body surface area during infancy or for weight only in newborns, whereas indexation for height should be preferred from 8–9 years onwards [16]. The majority of published studies define a cut-off value of ≥ 38.6 g/m^2.7^ for the presence of ventricular hypertrophy in childhood [24]. Recently reference centiles for left ventricular mass/m^2.7^ have been proposed that have been derived from 2273 normal weight subjects aged between 0 and 18 years [25].

Many data have been published concerning early arterial lesions in hypertensive children. The most precocious change of the arterial wall that can be shown by ultrasound techniques is thickening of the intima-media layers [26-30]. In the general pediatric population the intima-media thickness increases with age and is related to blood pressure [31]. Intima-media thickening develops in parallel with the development of left ventricular hypertrophy [26]. A prolonged exposure to increased blood pressure or to metabolic alterations may cause irreversible remodeling of the arterial walls. For this reason therapeutic interventions should be introduced as soon as possible in order to achieve regression of the vascular alterations as long as this is still achievable. It has been demonstrated that in hypertensive children the reduction of blood pressure values causes a regression of the carotid intima-media thickness [32]. The clinical relevance of intima-media thickness seems similar to that of echocardiographic measurement of left ventricular mass and the measurement of intima-media thickness could be used for the evaluation of organ damage and for the monitoring of the disease and its treatment. However such measurements should be standardized in order to avoid errors due to the use of different methods.

### Kidney

The kidney plays a central role in the development of many forms of hypertension. For this reason it is often difficult to determine the cause-effect relationship between increases in blood pressure and renal abnormalities. The prevalence of end-stage renal disease due to hypertension in adults is a well-known and worsening phenomenon. It is not entirely clear however to what extent rises in blood pressure contribute to the progression of renal alterations towards end-stage renal disease. Generally children and adolescents do not develop clinically evident renal abnormalities in response to increases in blood pressure. So the important question if there are any renal alterations in children with mild-moderate hypertension remains to be answered. The observation of contemporaneous hypertension and reduced glomerular filtration rate in children probably suggests the presence of a secondary form of hypertension. This also holds for the presence of proteinuria (>300 mg/day). The matter is more complex concerning microalbuminuria (30–300 mg/day, 2–30 mg/mmol urinary creatinine, 20–200 μg/min); in adult hypertension microalbuminuria is a consolidated marker of cardiovascular risk, whereas in children more studies would be necessary to define its significance in the presence of elevated blood pressure values. In pediatric subjects with chronic kidney disease it has been shown that high blood pressure and proteinuria are the two main predictive factors for progression to chronic renal failure [33]. To verify the renoprotective effect of antihypertensive treatment in children with chronic renal failure a European randomized multicenter trial has shown that in subjects with lower BP target (<50^th^ percentile) progression of renal failure was delayed more compared to subjects with higher BP target (50^th^-90^th^ percentile) [34].

### Nervous system

A reduction in baroreflex sensitivity has been shown in children with hypertension and high-normal blood pressure when compared to normotensive children [35]. While acknowledging that convulsions and cerebrovascular accidents in children may represent complications of some forms of severe or malignant hypertension, these complications are practically nonexistent in the primary forms of hypertension.

### Retina

Currently there are few data in literature regarding the effect of blood pressure on the retinal circulation in children. Fifty-one percent of hypertensive children are supposed to have retinal alterations that can be detected by direct ophthalmoscopy [36]. It has been revealed that in non-hypertensive children between 6 and 8 years of age each 10 mmHg increment in systolic blood pressure was associated with a reduction of the retinal arteriolar caliber of 1.43-2.08 micron, measured by quantitative analysis of the digital photographs of the retina [37] .

### Hypertensive emergencies

Hypertensive emergencies are defined as situations in which increases in blood pressure are accompanied by acute symptoms of organ damage: hypertensive encephalopathy (convulsions, cerebrovascular accidents) and congestive heart failure which expose the patients to life-threatening risks or to severe complications within minutes or hours. Hypertensive emergencies require immediate pharmacological treatment, while bearing in mind to avoid blood pressure reductions that are too abrupt. For this reason marked increases in blood pressure without symptoms of hypertensive encephalopathy or acute heart failure should be preferably treated with oral agents. As in children this condition is always due to secondary forms of hypertension specific tests should be performed [38-41].

### The role of the Family Pediatrician

The Italian Health System guarantees the assistance of the vast majority of children by a Family Pediatrician. This resource should allow the activation of prevention strategies for the most important chronic and degenerative diseases, starting immediately at young ages. Among these conditions hypertension plays a major role due to its high prevalence, especially when combined to overweight. For this reason the physician who follows children during the developmental age should:

•Collect an accurate family history to identify primary and secondary forms of hypertension.

•Use standardized methods and suitable instruments for a correct measurement of blood pressure in the child and interpret the values according to the most extensive and updated tables.

•Monitor blood pressure during annual control visits from the age of three.

•Repeat the blood pressure measurement on at least three different occasions when values are observed that could indicate hypertension or high normal blood pressure.

•Learn to make a first differential diagnosis between primary and secondary forms of hypertension on the basis of clinical history, physical examination, targeted examinations.

•Send patients with suspect secondary hypertension to referral centers.

•Apply the principles of the dietary and behavioral interventions in the treatment of the primary forms.

•Send patients with suspect secondary hypertension and cases of primary hypertension who do not respond to dietary and behavioral therapy to specialist centers.

•Cooperate with the specialist centers in the follow-up of the hypertensive child.

**Figure 2:**
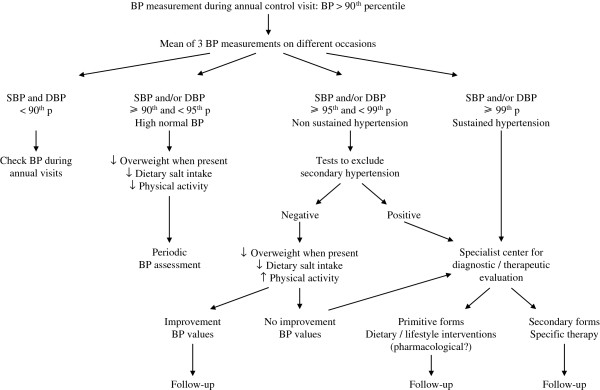
Flow chart for diagnosis and management of hypertension in children and adolescents.

National and international screening programs have identified a prevalence of 4% of children with high blood pressure [42], but the number of specialist centers for pediatric hypertension is limited. So it would be advisable to increase the availability and accessibility of these centers, but also to improve the expertise of the pediatricians in the management of subjects with high normal blood pressure or with non severe essential hypertension. For children with high normal blood pressure values it is sufficient to implement dietary and behavioral therapy and perform period blood pressure controls. For subjects with confirmed values above or equal to the 95^th^ percentile, there should be a distinction between those who have values above the 99^th^ percentile and those who do not present such high values (95-99^th^ percentile). In the first case it is suitable to send the patient to a specialist center because of the high probability of secondary hypertension, whereas in the second case the presence of peripheral pulses and negative results of simple diagnostic tests (blood sodium, potassium, creatinine, thyroid hormones, urine test) would direct towards the diagnosis of essential hypertension. In the latter condition, the Family Pediatrician can start dietary-behavioral therapy and send the child to a specialist center if no satisfactory improvement in blood pressure values is achieved. Once hypertension has been diagnosed it is advisable to search for other cardiovascular risk factors, such as total and HDL cholesterol, triglycerides, fasting glycemia and insulinemia. Figure 2 reports the algorithm that is suggested when elevated blood pressure values are found in children and adolescents. The hypertensive child and its parents should be followed with frequent control visits, because it is not easy to maintain treatment compliance in a disease that does not entail subjective disturbances. It would be desirable that in the future the network of Family Pediatricians may collaborate in the collection of epidemiological data on hypertension in the developmental age.

### Role of specialist centers

The specialist centers have the duty to guarantee a multidisciplinary approach to the hypertension problem in children. They should provide pediatric, cardiologic, nephrologic, endocrinologic, dietary and in some cases psychological expertise. These centers should obtain ample experience in the evaluation of organ damage, interpretation of 24 hour ambulatory blood pressure monitoring and self-measurement of blood pressure at home. They should also have access to laboratory techniques and instruments necessary for the diagnosis of different forms of secondary hypertension. It is important that the specialist centers build communication channels between pediatricians and family doctors with the aim of outlining the therapy and monitoring the hypertensive child.

### Prevention of hypertension in children and adolescents

Prevention should aim at avoiding the onset of hypertension both in children and in adults. Therefore the pediatrician should identify the main risk factors:

•Family history of hypertension

A lot of international studies show that children coming from hypertensive families have a greater risk to present with elevated blood pressure values [7,8].

•Low birth weight

Epidemiological studies have shown that intrauterine growth delay and low birth weight are risk factors for hypertension and cardiovascular diseases in adulthood [43,44]. A large meta-analysis on 55 studies has revealed an inverse relationship between birth weight and blood pressure values [45]. The hypothesis of a fetal origin of diseases that become manifest in adult age suggests that environmental factors and prenatal nutritional factors in particular act during the early phases of life predisposing to metabolic and cardiovascular diseases [46]. Experimental studies demonstrate that placental insufficiency modifies myocardiocyte and coronary maturation of the fetus [47]. Ultrasound studies performed in pregnant women show that intrauterine growth delay induces alterations in the fetal cardiovascular system [48,49]. The relationship between poor intrauterine growth and hypertension may be explained by different mechanisms such as development of a lower number of nephrons, excessive exposure to glucocorticoids, changes in the regulation of the renin-angiotensin-aldosterone system and early development of insulin resistance [46]. Rapid postnatal weight recovery and overweight in later years may play an important role in these subjects [50]. As there are no specific therapies available than can modify the effects initiated during intrauterine life, the only remaining treatment is prevention. The inverse relationship between birth weight and blood pressure becomes more marked with increasing age. Children with low birth weight should have their blood pressure monitored throughout childhood. Special attention should be paid to their nutrition program by promoting breastfeeding and recommending weaning and nutrition programs that avoid excessively rapid or intense weight gain. More than other children these subjects should be recommended to limit salt intake and to increase their physical activity.

•Overweight and obesity

Overweight represents over 50% of all the causes of hypertension in children [51]. The relationship between BMI and blood pressure values has been extensively documented. Recently a correlation with other markers of obesity, particularly abdominal obesity, has been found [52].

•Sedentary behavior

Adequate physical activity lasting 40 minutes for 3–5 times a week causes a reduction in blood pressure values [51,53]. A dual intervention on both diet and physical activity contributes even more to blood pressure reduction in children [54].

•Substances that increase blood pressure

Salt rich diets already affect blood pressure in childhood [55], while potassium supplementations could reduce blood pressure values [56].

Some drugs increase blood pressure (steroids, erythropoietin, theophylline, beta-stimulants, cyclosporine, tacrolimus, tricyclic antidepressants, antipsychotics, nasal decongestionants, oral contraceptives). Alcohol and excessive licorice consumption may increase blood pressure. Some psychoactive drugs and particularly cocaine and amphetamines raise blood pressure as well.

•Cigarette smoke

Infants whose mothers smoked during pregnancy have higher blood pressure than babies born from non-smoking mothers [57] and they show an increased hypertensive response to stress compared to peers up to the age of one year [58]. Exposure to passive smoking as well seems to play a role in increasing blood pressure [59,60].

•Non sustained form of hypertension

The finding of elevated blood pressure values at a control visit that is not confirmed at subsequent measurements could represent a risk factor for later development of hypertension [61]. A recent study reports that subjects with elevated blood pressure values at in the doctor’s office and normal range values at 24 hour ambulatory blood pressure monitoring presented greater cardiac masses than normotensives of the same age [62].

Table 7 summarizes the information that should be collected in the case of high blood pressure findings in children or adolescents.

**Table 7:** Medical history to record in children and adolescents with hypertension

	
** Family history**	
•	Hypertension
•	Cardiovascular and cerebrovascular disease
•	Diabetes mellitus
•	Dyslipidemia
•	Obesity
•	Hereditary renal disease (Polycystic kidney disease)
•	Hereditary endocrine disease (pheochromocytoma, adrenal hyperplasia, multiple endocrine neoplasia , von Hippel-Lindau)
•	Syndromes associated with hypertension (neurofibromatosis)
** Clinical history**	
•	**Perinatal history**: birth weight, gestational age, oligohydramnios, anoxia, umbilical artery catheterization
•	**Previous history**: urinary tract infection, renal or urological disease, cardiac, endocrine (including diabetes) or neurological disease, growth retardation
•	**Symptoms suggestive of secondary hypertension**: dysuria, thirst/polyuria, nocturia, hematuria, edema, weight loss, failure to thrive, palpitations, sweating, fever, pallor, flushing, cold extremities, intermittent claudication, virilization, primary amenorrhea, male pseudohermaphroditism
•	**Symptoms suggestive of target organ damage**: headache, epistaxis, vertigo, visual impairment, facial palsy, fits, strokes, dyspnea
•	**Sleep history**: snoring, apnea, daytime somnolence
•	**Risk factor history**: low physical exercise level, incorrect dietary habits, smoking, alcohol, licorice
•	**Drug intake**: cyclosporine, tacrolimus, tricyclic anti-depressants, antipsychotics, decongestants, oral contraceptives, illegal drugs
•	**Pregnancy**

Lurbe et al. “Management of High Blood Pressure in Children and Adolescents: recommendations of the ESH”. *Journal of Hypertension* 2009 [2] (modified).

### Newborn hypertension

The first blood pressure measurements in newborns with reliable techniques go back to the 1970’s. In those years Menghetti published mean systolic blood pressure values in 6.000 Italian full-term newborns between 43.1 mmHg on the first day and 62.0 mmHg after 6 days of life using ultrasound techniques [63]. Generally in preterm newborns values are about 10 mmHg lower. The recent availability of oscillometric devices opens new study possibilities. From the data in literature it can be affirmed that based on both Doppler and oscillometric evaluations a diagnosis of neonatal hypertension can be made in full-term newborns when observing systolic blood pressure values that are permanently higher than 90 mmHg [64]. Correct recordings of the blood pressure in newborns require cuffs that have appropriate length (at least 3/2 the circumference of the arm) and width (about 2/3 the length of the arm). As a rule the cuff size used in preterm babies is 2.5 cm, in full-term newborns 4 cm, and 6–8 cm in 1 month old babies [65]. In order to obtain correct blood pressure recordings the newborn/baby should be calm and supine and the mean of 3 measurements should be considered. The prevalence of neonatal hypertension has been estimated between 0.2 and 3% in the newborn population as whole, whereas in preterm and full-term infants cared for in neonatal intensive care units hypertension is observed in more than 2% of the subjects. These data should be confirmed by systematic measurements in all birth centers and hospitals and the blood pressure values should be reported in the hospital discharge papers. Hypertension in newborns and infants is almost exclusively seen in the secondary form. The main causes involve either the renovascular system with vascular stenosis or thrombosis, the cardiovascular system with aortic coarctation, or the intrinsic renal forms (hydronephrosis, polycystic kidney, hypoplastic kidney, Wilms tumor and pyelonephritis) and the endocrine forms (neuroblastoma, adrenogenital syndrome and pheochromocytoma) [66]. Umbilical artery catheterization with consequent thromboembolism of the aorta and/or the renal arteries is one of the most typical iatrogenic causes in the neonatal intensive care units. The hypertensive newborn may present irritability, seizures, respiratory disturbances and acute heart failure. First-line laboratory test are: urine test, serum ionogram, blood count, blood urea nitrogen and creatinine. Catecholamine and urinary 17-ketosteroid dosing may be useful. Diagnostic imaging may involve echography, urography and even aortography when necessary. For the prevention of hypertension breastfeeding should be promoted which should be exclusive during the first 6 months and maintained up to 1 year [67]. If breast milk is not available it is advised to use low sodium and low protein formulations. During weaning no salt should be added to the baby food.

### Secondary forms of hypertension

Hypertension is defined secondary when causes can be found that can be treated with specific interventions. The causes of hypertension vary according to different periods in childhood. Secondary hypertension is more frequent during the first years of life whereas the prevalence of essential hypertension increases with age. The attention that has to be given in finding secondary causes of hypertension should be inversely proportional to age and directly proportional to the severity of the hypertension. The evaluation of children with hypertension should always be aimed at excluding secondary forms. In children hypertension due to renal causes (chronic kidney disease or renovascular hypertension) and aortic coarctation accounts for 70 to 90% of the secondary forms [68,69]. Endocrinologic (Primary hyperaldosteronism, Cushing's syndrome, Adrenogenital syndrome, Hyperthyroidism, Pheochromocytoma) and other causes (drug-induced and genetic forms) are more uncommon. The assessment of a hypertensive child should consider blood pressure values, age, clinical signs and family history. Table 8 shows the guiding criteria for the distinction between essential and secondary forms of hypertension. A detailed description of the diagnostic processes of secondary forms of hypertension [70-72] goes beyond the goals of the present recommendations.

**Table 8:** Guiding criteria for the differential diagnosis between essential hypertension and secondary forms of hypertension

	**Essential forms**	**Secondary forms**
Onset	Most frequent in children and adolescents	Often early
Discovery	Casual during annual control visit	Often underlying disease already known
Blood pressure values	Moderately elevated	Often markedly elevated
Associated symptoms	None	According to specific disorder
Family history	Often positive for essential hypertension	Familiar forms are rare
Overweight	Often present	Not frequent
Femoral pulse	Present	Reduced or absent with coarctation of the aorta
Difference between BP values in upper and lower extremities	Not present	Present with coarctation of the aorta
Blood sodium, potassium and creatinine levels, urinalysis, thyroid hormones	Normal	Altered in some specific disorders
Echocardiography	Normal (left ventricular hypertrophy may be present)	Allows diagnosis of coarctation of the aorta (left ventricular hypertrophy may be present)

### Dietary-behavioral interventions

Dietary-behavioral therapy is indicated in children with hypertension or high-normal blood pressure and should be advised to those with transiently elevated blood pressure values or with positive family history for hypertension. This therapy should be maintained even if pharmacological treatment is adopted as well [73-75]. Non-pharmacological treatment of hypertension is based on correct lifestyle and nutrition; participation of the parents in the acceptance and execution of the treatment is important for increasing the adhesion to the program. Dietary-behavioral therapy is based on the following points:

•Reduction of overweight

As increased blood pressure is often associated with overweight [5,76,77], weight reduction can reduce blood pressure levels. Regarding the definition of overweight and obesity in childhood the criteria proposed by Cole in 2000 [78] need to mentioned; these criteria have been adopted by the International Obesity Task Force and use body mass index (BMI = weight in kg/height^2^ in m) depending on age and gender. Goals of the treatment are: maintaining a normal height-weight growth in normal weight children; trying to improve BMI by a greater increase in height compared to weight in overweight subjects up to 8 years of age; pursuing gradual weight loss in older children and adolescents with excess weight. In obese hypertensive subjects the aim is achieving weight loss, 1–2 kg a month in adolescents, through the adoption of a moderately hypocaloric diet. In all cases varied dietary schedules are advised which include all food groups and offer a correct division of the meals (15% breakfast, 5% snack, 40% lunch, 10% snack, 30% dinner), having the best possible distribution of caloric (proteins 10-12%, lipids 28-30%, carbohydrates 60%) and non-caloric nutrients (vitamins, minerals, oligoelements, fibers) according to the indications by LARN [79] and observing the nutritional needs of growing individuals [80]. In the case of severe obesity (BMI > 99^th^ percentile according to the parameters of the American Academy of Pediatrics [81]), the dietary-behavioral interventions should be individualized by an integrated multidisciplinary team.

•Reduction of salt intake

Trials on the relationship between sodium intake and blood pressure in children are limited. In adults it has been demonstrated that moderate salt intake restriction reduces blood pressure in sodium-sensitive subjects, i.e. 50-60% of all hypertensives [55]. A meta-analysis has shown that a modest reduction in sodium intake in children causes a decrease in blood pressure values and may determine a reduction of the physiological age-related increase in blood pressure [82]. Currently only few data are available on sodium intake in young generations. The rise in use of precooked foods and outdoor eating may have increased salt intake compared to the past. Dietary salt can be divided in discretionary salt which is kitchen salt added during and after cooking and at the table (about 36%) and non-discretionary salt. Non-discretionary salt includes the salt that is present in the food itself (about 10%) and the salt that has been added for preservation and increasing taste of the food (remaining 54%) [83]. People have an innate tendency to appreciate salty flavor but the amount of salt needed by every single person depends on the dietary habits learned during childhood. In order to limit sodium intake it is advised to eat almost exclusively fresh food and to cook it without salt thus reducing the use of NaCl to a level teaspoon full equaling 5 g of salt or approximately 2 g of sodium. In this way daily intake can be limited to about 2.5 g or even less after further discretionary salt reduction. It would be advisable to use unsalted bread, like Tuscan bread. The consumption of fruit and vegetables is particularly recommended in hypertension as, besides limiting caloric intake and favoring sufficient amounts of vitamins and micronutrients, it also increases the intake of potassium which is believed to have a positive effect on blood pressure [56]. Sufficient calcium intake is recommended that can be obtained by consumption of nonfat milk and dairy products.

•Increase in physical activity

Forty minutes of aerobic-based physical activity 3–5 days/week are required to improve cardiovascular function and reduce blood pressure in children [51]. It is also necessary to avoid more than 2 h daily of sedentary activities [2]. Regular sport practice should be encouraged (excluding particularly competitive activities in children with stage 2 hypertension that cannot be controlled with pharmacological therapy) as well as unstructured physical activity [74,84] such as walking to school, taking a walk, riding a bike, playing. Outdoor activities should be stimulated, whereas time spent in sedentary activities such as watching TV, playing videogames, suing the computer should be limited [85]. A research by the Italian Society of Pediatrics shows that 60% of 12–14 year old children spend 1–3 hours a day watching TV and 20% even exceed 3 hours [86]. Fifty-seven percent have a television set and 50% have a computer in their own bedroom and this, besides increasing the use of these appliances [87], also reduces the parents’ control of the programs that are transmitted. Television programs are interrupted by many TV commercials that often propose highly caloric and salty foods. Therefore children who spend too much time watching TV may also be those who present the worst nutritional habits [88,89]. Schools are also responsible for the scarce physical activity of children and adolescents. In addition to school hours with little sport activities much time is spent doing homework. Furthermore there is little space in school programs for promoting healthy lifestyle and nutrition. Table 9 summarizes the measures to be taken in the case of hypertension in children. Even though multiple and coordinated awareness campaigns aiming at a correct lifestyle are advisable, the child’s family still remains the main environment for teaching the right habits especially through the parents’ example. The detection of increased blood pressure levels in a child may stimulate the improvement of the entire family’s lifestyle. Whereas parents and teachers usually are receptive to the message that healthy lifestyle and nutrition prevent chronic degenerative diseases, young people do not show much, if any, interest in this kind of approach. Other motivations should be emphasized such as physical fitness, good looks, improvement of school and sport performances, all obtainable by healthy dietary and behavioral habits. It should be the task of the Family Pediatrician to convey these messages in an appropriate and balanced manner.

**Table 9:** Lifestyle and dietary recommendations to reduce high blood pressure values

	
**Goals**	
•	**BMI in the normal weight range**: maintain BMI to prevent overweight
•	**BMI in the overweight range**: weight maintenance in younger children or gradual weight loss in older children and adolescents to return to the normal weight range
•	**BMI in the obesity range**: gradual weight loss (1–2 kg/month) to achieve normal weight
**General recommendations**	
•	Moderate to vigorous physical aerobic activity for 40 min, 3–5 days/week and avoid more than 2 h of daily sedentary activities (besides school hours)
•	Participation in (competitive) sports activities limited only in the presence of uncontrolled stage 2 hypertension
•	Avoid severe dietary restrictions, reduce portion size, stimulate the habit of having breakfast
•	Limit salt intake
•	Avoid intake of excess sugar, excess soft drinks, saturated and trans fat, animal protein
•	Drink water
•	Stimulate the intake of healthy food (fruit, vegetables, legumes, whole grain products, fish)
•	Implement the behavioral changes (physical activity and diet) suitable for individual and family characteristics
•	Establish realistic goals
•	Involve the family, caregivers, teachers and other educators in the process of dietary and life-style changes
•	Provide educational support and materials
•	Develop reward systems (non-food) to achieve healthy behavior

Lurbe et al. “Management of High Blood Pressure in Children and Adolescents: recommendations of the ESH”. *Journal of Hypertension* 2009 [2] (modified).

### Pharmacological therapy

Pharmacological therapy, when needed, should not in any condition exclude dietary and behavioral therapy as the latter may allow dose reduction of drugs, better therapeutic control and more effective prevention of other cardiovascular risk factors.

Drug treatment should be started in hypertensive children with organ damage or kidney disease when blood pressure values exceed the limits indicated in the treatment goals (see Table 10). As univocal recommendations on the pharmacological treatment of primary hypertension in subjects without organ damage are not available, each of these conditions should be evaluated individually. Pharmacological treatment is needed in the presence of blood pressure values that remain above the 95^th^ percentile unless non-pharmacological interventions and in the case of initial organ damage (particularly increase in left ventricular mass). Drug therapy should be considered in the presence of severe obesity with concomitant diseases, and may later on be interrupted following a positive response to the dietary-behavioral interventions. The drugs that are currently recommended for the treatment of hypertension in adults are also prescribed for children and adolescents (Table 11): a) renin-angiotensin-aldosterone system blockers, ACE-inhibitors [90-92] and AT_1_-receptor antagonists, sartans [93-95] (aliskiren, a direct renin inhibitor has not been evaluated in pediatric studies yet); b) beta-blockers [96]; c) calcium-antagonists [97]; d) diuretics [98]. Both renin-angiotensin-aldosterone system blockers and beta-blockers reduce blood pressure by inactivation of the renin-angiotensin-aldosterone system and by reducing peripheral vascular resistance (R drugs). Calcium antagonists exert a direct vasodilatory effect, while diuretics increase natriuresis and reduce blood volume (V drugs). In subjects with preserved renal function the blood pressure lowering efficacy is practically identical for renin-angiotensin-aldosterone system blockers, beta-blockers, calcium antagonists and diuretics. In diabetics and in the presence of kidney disease, especially with pathological proteinuria, renin-angiotensin-aldosterone system blockers should be preferred [99,100].

**Table 10:** Therapeutic management of hypertension

	
**Evidence in favor of therapeutic management**	
•	Reduce mortality and sequelae in the long-term
•	Reduce left ventricular hypertrophy
•	Reduce urinary albumin excretion
•	Reduce rate of progression to end-stage renal disease
**When to start antihypertensive treatment**	
•	Non-pharmacological therapy should be initiated in all children with high normal blood pressure or hypertension
•	Non-pharmacological therapy should be continued after starting pharmacological therapy
•	Pharmacological therapy should be initiated when patients have symptomatic hypertension, target organ damage, secondary hypertension or diabetes mellitus type 1 or 2
•	Pharmacological therapy should be considered in the presence of clear increases in blood pressure levels or in the case of severe obesity with associated clinical conditions. Pharmacological therapy may be interrupted following positive results with lifestyle and dietary changes
**BP targets**	
•	In general: blood pressure below the 90^th^ percentile, specific for age, sex and height
•	Chronic kidney disease: blood pressure below the 75^th^ percentile without proteinuria and below the 50^th^ percentile in cases of proteinuria (urine total protein creatinine ratio >0.20 mg/mg)

Lurbe et al. “Management of High Blood Pressure in Children and Adolescents: recommendations of the ESH”. *Journal of Hypertension* 2009 [2] (modified).

**Table 11:** Drugs used for the treatment of hypertension in children and adolescents, which on the basis of their long half-life are administered once-a-day

		**Recommended Daily Dose (mg)**		
		**Body Weight (kg)**		
**Class**	**Drug name**	**10-25**	**25-40**	**>40**
Renin-Angiotensin-Aldosterone				
System Blockers				
AT1-receptor Blockers	Candesartan	4-8	8-16	16-32
	Irbesartan	37-75	75-150	150-300
	Losartan	12-25	(25)-100	(50)-100
	Olmesartan	(2.5)-10	5-20	20-40
	Valsartan	20-40	40-80	80-160
ACE -Inhibitors	Benazepril	2.5-5.0	5.0-10	10-20 [40]
	Fosinopril	1.3-2.5	2.5-10	5.0-20 [40]
	Lisinopril	2.5-10	5.0-20	10.30 [40]
	Quinapril	2.5-5.0	5.0-10	10-20 [40]
	Ramipril	1.3-2.5	2.5-10	5.0-20
Beta-Blockers	Atenolol	12-25	25-100	100-200
	Bisoprolol	1.2-2.5	2.5-5.0	5.0-10
	Metoprolol	10-25	25-100	100-200
Calcium Antagonists	Amlodipine	2.5-5.0	5-10	10-20
Thiazide Diuretics	Chlorthalidone	6-12	12-25	25-50
	Hydrochlorothiazide	6-12	12-25	25-50

Retard formulations are unsuitable as they are often badly absorbed in children (and when split in two the prolonged effect of these formulations disappears).

In practice it would be advisable to use drugs with prolonged effects (retard formulations should be avoided as they are badly absorbed by children and loose their prolonged effect once the tablets are split) and also to prescribe drugs with the least side effects. Adverse side effects are most frequent with diuretics, followed by beta-blockers, calcium antagonists, ACE-inhibitors and finally sartans. Almost none of the drugs used for treating hypertension has specific pediatric formulations. When tablets are divided or pulverized for use in children they often assume a distasteful flavor. The thiazide diuretics hydrchlorothiazide and chlorthalidone, the calcium antagonist lercanidipine and the AT_1_-receptor blocker candesartan do not have any flavor and therefore they can easily be administered in little children. Treatment should start with a single drug. If the blood pressure goal is not reached within 4–8 weeks the dose should be increased or a second drug should be added. Not all drug combinations are rational: an R drug (renin-angiotensin-aldosterone system blocker or beta-blocker) can reasonably be associated with a V drug (calcium antagonist or diuretic). In clinical practice fixed drug combinations are often used, the most frequently employed combination being an ACE-inhibitor plus a thiazide diuretic. The intervention of a specialist is always necessary when the patient weighs less than 10–15 kg, when the blood pressure goal is not reached after two drug treatment and in the presence of impaired renal function (in this situation thiazide diuretics are ineffective and should be replaced by loop diuretics such as furosemide).

## Conclusions

The present document endorses the recent recommendations of the European Society of Hypertension and represents their translation into the Italian clinical reality. Two specific aspects characterize our country regarding the hypertension problem among the pediatric population. On the one hand Italy, and especially the southern regions, is one of the leading European countries for prevalence of overweight. On the other hand there is a widespread organization of the primary health care of children and adolescents in the whole country. The increase of excess weight in children and adolescents may cause a rise in hypertension and other risk factors that are associated with overweight and obesity. The important increase of cardiovascular diseases that will arise once the present young generations will become adults is of great concern. Sufficient resources could become available to avert this phenomenon, provided that the entire society together with physicians and pediatricians in particular become aware of the severity and the urgency of the problem. For this reason preventive strategies such as promoting healthy lifestyle and nutrition as well as limiting salt and alcohol intake and quitting cigarette smoking are necessary. However, these general measures should be assisted by procedures aiming at revealing specific and individual cardiovascular risk factors. Blood pressure measurement of children and adolescents is definitely the most simple and economic of these procedures. This practice will reveal an important number of subjects with increased blood pressure values thus stimulating both the institutions that take care of children’s health (families, schools, societies, political decision makers at different levels) and scientific research workers. According to the recommendations of the European Society of Hypertension [2], the future goals are: The efforts and investments in the field of prevention and treatment of hypertension and of cardiovascular risk in general, when started in childhood, may have a very positive effect both on health and on saving of economic resources.

•Development of accurate non-mercury sphygmomanometer for auscultatory blood pressure measurement and accurate automatic devices for oscillometric blood pressure measurement and careful comparison of the two methods in children and adolescents

•Implementation of reference values for office, 24h ambulatory and self-measured blood pressure in children and adolescents

•Increase the knowledge of the use of out-of-office blood pressure measuring systems in children and adolescents

•Collect data on early organ damage in order to allow risk stratification and to define treatment goals

•Conduct large, long-term randomized therapeutic trials on the onset of early organ damage (microalbuminuria and/or left ventricular hypertrophy) to obtain information about when to start pharmacological therapy and about the blood pressure goals to achieve

•Conduct controlled studies to define the advantages and disadvantages of antihypertensive treatment and establish adequate doses in children and adolescents

## Competing interests

All authors declared that they have no competing interests.

## Authors’ contributions

All authors have been involved in drafting the manuscript and revising it critically for intellectual content and have given final approval of the version to be published. All authors read and approved the final manuscript.
